# Editorial: Peri-implantitis management: exploring multifactorial etiology and novel treatment modalities

**DOI:** 10.3389/fdmed.2025.1754247

**Published:** 2026-01-19

**Authors:** Catherine Giannopoulou, Selena Toma, Jérôme Frédéric Lasserre

**Affiliations:** 1 Division of Regenerative Dental Medicine and Periodontology, University Clinics of Dental Medicine, University of Geneva, Geneva, Switzerland; 2 Department of Orthodontics and Periodontology, Institut de Médecine Dentaire et de Stomatologie, Cliniques Universitaires Saint Luc, Université catholique de Louvain, Brussels, Belgium

**Keywords:** explantation, implant surface, maintenance, peri-Implantitis, photodynamic therapy, platelet-rich fibrin, residual bone

Editorial on the Research Topic Peri-implantitis management: exploring multifactorial etiology and novel treatment modalities

Peri-implantitis remains one of the most challenging complications in implant dentistry, reflecting a complex interplay between microbial, host-related, and implant-specific factors. As implant therapy becomes increasingly common, the need for effective, predictable, and evidence-based peri-implantitis management has never been greater.

The current research topic synthesizes recent advances and ongoing debates in peri-implantitis management yet also exposes critical gaps that continue to hinder clinical decision-making.

The research topic includes two systematic reviews, two other reviews and a prospective case series study.

A central point emerging from recent evidence is the role of implant surface characteristics in long-term treatment outcomes. The systematic review by Gardelis et al. on surface modifications demonstrates that roughened implants—despite their well-established osseointegration advantages—exhibit higher recurrence rates and increased implant loss following surgical peri-implantitis treatment compared with machined surfaces. Outcomes of reconstructive surgical approaches appear more consistent, particularly when combined with grafting materials and membranes. Still, methodological heterogeneity limits the strength of these conclusions, underscoring the need for well-controlled longitudinal studies.

Beyond surgery, growing attention has been directed toward minimally invasive and adjunctive therapies. Photodynamic therapy (PDT) represents one of the most thoroughly investigated options. The comprehensive meta-analysis of Yan et al. shows that when combined with mechanical debridement, PDT consistently improves probing depth, bleeding on probing, and crestal bone stability—both in mucositis and peri-implantitis. Nevertheless, the moderate-to-low certainty of evidence and frequent methodological bias call for cautious interpretation. PDT is emerging as a valuable adjunct, but not a standalone solution.

The biological environment also plays a crucial prognostic role. Evidence from narrative reviews highlights that residual bone level is an essential determinant for surgical planning. As reported by Martin-Cabezas and Giannopoulou when bone loss exceeds 50% of implant length, treatment success drops dramatically, and explantation often becomes the most predictable option. This aligns with the broader recognition that peri-implantitis cannot be uniformly managed; instead, clinicians must integrate defect morphology, implant characteristics, and systemic factors into individualized treatment plans.

Innovative regenerative adjuncts such as injectable platelet-rich fibrin (i-PRF) were explored by Deterville et al. who conducted a prospective case series. Their results suggested that the combination of glycine air polishing and i-PRF can significantly reduce inflammation, improve clinical parameters, and stabilize bone levels over six months. Yet despite these encouraging changes, complete disease resolution—defined by absence of bleeding on probing alongside with bone stability—was not achieved, illustrating the inherent difficulty in halting peri-implant inflammation through nonsurgical means alone.

Finally, disease prevention remains the cornerstone of long-term implant success. Recent clinical guidelines emphasize rigorous and individualized professional maintenance, but the lack of randomized clinical trials leaves many recommendations based on expert opinion rather than high-level evidence. In their review, Sahrmann and Giannopoulou emphasized that regular monitoring of probing depths, bleeding, plaque control, and radiographic changes is essential, but consensus is still lacking on optimal recall intervals and specific diagnostic thresholds.

Collectively, these five articles reaffirm that peri-implantitis is multifactorial in origin and multifaceted in its management. Research gaps are identified and summarized:

Lack of Standardized Diagnostic and Prognostic Criteria: There is no universally accepted threshold for “advanced” bone loss, complicating treatment decisions and prognostication (Martin-Cabezas and Giannopoulou).
Limited High-Quality Evidence for Adjunctive Therapies: While modalities like i-PRF and PDT show promise, their long-term efficacy and optimal protocols remain to be established through rigorous randomized controlled trials (Deterville et al.; Yan et al.).
Uncertainty Regarding Implant Surface Modifications: The impact of surface characteristics on long-term outcomes is not fully understood, necessitating further research (Gardelis et al.).
Insufficient Data on Maintenance Protocols: There is a dearth of randomized clinical trials guiding specific diagnostic steps and treatment decisions during recall sessions (Sahrmann and Giannopoulou).

Improved understanding of prognostic indicators, refinement of surgical protocols, development of adjunctive therapies, and strengthening of preventive strategies remain crucial.

